# The influence of antibiotic-loaded cement spacers on the risk of reinfection after septic two-stage hip revision surgery

**DOI:** 10.1007/s15010-017-1081-5

**Published:** 2017-10-24

**Authors:** Kevin Staats, Florian Sevelda, Alexandra Kaider, Christoph Böhler, Irene K. Sigmund, Stephan E. Puchner, Reinhard Windhager, Johannes Holinka

**Affiliations:** 1 Department of Orthopaedic Surgery, Medical University of Vienna, Waehringer Guertel 18-20, 1090 Vienna, Austria; 2 Section for Clinical Biometrics, Centre for Medical Statistics, Informatics, and Intelligent Systems, Medical University of Vienna, Waehringer Guertel 18-20, 1090 Vienna, Austria

**Keywords:** Periprosthetic joint infection, Hip, Spacer

## Abstract

**Purpose:**

The aim of this study was the evaluation of possible outcome differences of patients undergoing two-stage hip exchange with antibiotic-loaded spacers, compared to patients without an interim spacer implantation.

**Methods:**

We evaluated 46 patients undergoing two-stage hip revision surgery. Twenty-five patients received an interim ALS. Additional to a Kaplan–Meier survival analysis, a competing risk analysis was performed to estimate the cumulative incidence function for re-revisions due to infection accounting for death as a competing event.

**Results:**

Nine patients (seven non-ALS vs. two ALS) had to undergo re-revision surgery due to reinfection of the hip joint. The non-ALS group showed a risk of re-revision of 19% (95% CI 5–38%) at 12 and 24 months and 30% (95% CI 12–51%) at 36 months. The group with ALS implantation displayed a 0% risk of re-revision surgery in the first 36 months. The Gray test revealed a significant difference in the cumulative incidence between both observed groups (*p* = 0.026).

**Conclusion:**

Our findings suggest that ALS implantation significantly reduces the risk of reinfection after two-stage hip revision surgery.

## Introduction

Periprosthetic joint infection (PJI) is one of the most demanding complications after total hip arthroplasty (THA), it has a vast impact on patient’s morbidity and mortality, and remains a socio-economic problem [1–4]. The best therapeutic strategy for PJI still remains open to widespread debate [1, 5–9]. Two-stage revisions show satisfying success rates, at around 90%, and therefore, represent the standard procedure especially for late/chronic infections [6, 9, 10]. During the first-stage procedure, the infected prosthesis is removed and an implantation of an interim antibiotic-loaded cement spacer (ALS) can be considered. The major advantage of the usage of ALS is the possibility of maintaining high antibiotic concentrations that reach a local therapeutic level [11]. Therefore, ALS may contribute to the eradication of a PJI, as ALS improves the antimicrobial efficacy of systemic antibiotics [12]. An additional benefit may be the avoidance of possible contractures through the reduction of the dead space after explantation of the infected implants [6, 13].

Despite the broad usage of ALS in two-stage revisions, there still remains concern in regard to induction of resistances and development of biofilm-forming microorganisms [14–17]. In the worst-case scenario, the spacer itself becomes—due to its surface conditions—a vehicle for adherent microorganisms [12, 18]. Zimmerli et al. claim that only in cases where no “difficult-to-treat”(DTT)-microorganisms (MRSA, small colony variants of staphylococci, enterococci, quinolone-resistant *Pseudomonas aeruginosa* and fungi) are isolated, should an ALS be implanted during the first-stage procedure [19]. Additionally, with the use of rather new diagnostic tools, spacers seem to be more colonized by microorganisms than priorly expected [20, 21].

We, therefore, raised the question of whether the treatment of ALS has an effect on the re-revision rate and risk of reinfection in patients undergoing a two-stage procedure due to PJI in THA. We hypothesized that ALS has a benefit on the outcome parameters and cause a reduction in the reinfection rate, regardless of the type of microorganism.

## Patients and methods

We performed a retrospective study of patients suffering from a deep infection after total hip arthroplasty (THA) between 2001 and 2014 using the hospital database. The local ethics committee approved the study.

PJI was suspected preoperatively by the presence of leucocytosis, elevated C-reactive protein-levels (CRP), pain, swelling, local erythema and warmth. PJI was verified intraoperatively through positive microbiology and/or positive histopathological examination. Upon examining positive culture results, so-called “difficult-to-treat” microorganisms were registered. These DTT microorganisms involved Methicillin-resistant *Staphylococcus aureus* (MRSA), small colony variants of staphylococci, enterococci, quinolone resistant *P. aeruginosa* and fungi. [19, 22].

**Fig. 1:**
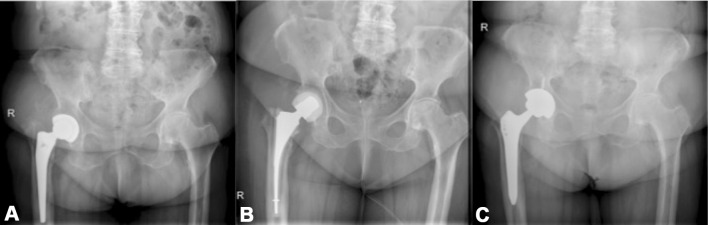
Example of a female patient with hip arthroplasty of the right hip joint (a) who had undergone two-stage revision surgery due to periprosthetic joint infection. Interim antibiotic-loaded spacer was implanted between explantation (b) and reimplantation (c)

**Fig. 2:**
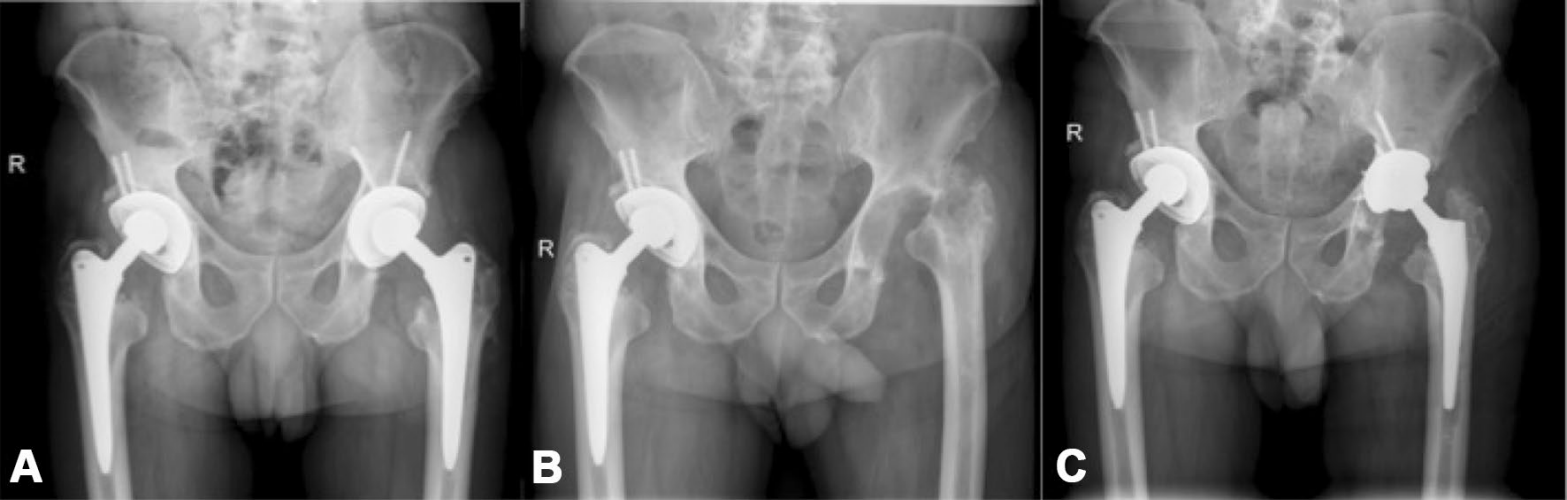
Example of a male patient with bilateral hip arthroplasty (a) who had undergone two-stage revision surgery due to periprosthetic joint infection on the left side. No interim antibiotic-loaded spacer was implanted between explantation (b) and reimplantation (c)

46 patients (21 female and 25 male, mean age = 64 years, range 24–87 years) were included in this study. Twenty-one patients underwent two-stage revision without an interim ALS (example Fig. 1) and 25 patients received an ALS after the first stage (example Fig. 2). No randomization was performed, regardless of whether patients were provided with an ALS or without ALS implantation. ALS usage was dependent solely upon the surgeon’s choice.

For all ALSs included, the same antibiotic bone cement, containing 0.5 g of Gentamicin and 2 g of Vancomycin per 40 g bone cement (COPAL^®^ G+V 40, Heraeus, Wehrheim, Germany) was used. In the ALS group a custom-moulded articulating spacer system (Stage One^®^, Biomet, Warsaw, USA) was implanted. All patients received intravenously administered antibiotics 30 min prior to surgery and for at least 4 weeks postoperatively with 10–14 days iv-administration. If a pathogen was detected, the treatment was then assessed following performance of an antibiogram and a resistogram and adjusted accordingly if required. Treatment adjustments were performed using the antibiotic treatment recommendations by Trampuz and Zimmerli [19]. If patients were discharged before reimplantation, the antibiotics were switched to orally administered options in accordance with prior consultation with our specialist for infectious diseases. Reimplantation was planned following a minimum of 4 weeks from the date of explantation. During this inter-stage period, CRP and leucocyte levels were examined on a regular basis. After reimplantation, all patients received iv-antibiotics for at least 7 days, which were then changed to oral antibiotics. These antibiotics were then administered for a period of time ranging from 21 days up to 5 weeks.

Since patient-specific comorbidities predispose for periprosthetic infections [23], systemic host factors and infection types were evaluated using the scoring system described by McPherson et al. [8]. Patients with no compromising host factors were classified as systemic host grade A, patients with one or two compromising factors were assigned to systemic host grade B and patients with three or more compromising factors were classified as systemic host grade C. Table 1 exemplifies those compromising host factors. This classification system also distinguishes between early infections occurring in the first 4 weeks after primary implantation, haematogenous infection also occurring in the first 4 weeks but usually with previous well-functioning joint and presence of bacteremia and late (chronic) infection with symptoms occuring after 4 weeks after primary implantation. Table 2 displays the detected pathogens and inflammatory parameters during initial infection for both groups.

**Table 1:** Compromising host factors following the grading system by McPherson et al. [8]

Age ≥ 80 years
Alcoholism
Chronic active dermatitis or cellulitis
Chronic indwelling catheter
Chronic malnutrition (albumin ≤ 3.0 g/dl)
Current nicotine use (inhalational or oral)
Diabetes (requiring oral agents or/and insulin)
Hepatic insufficiency (cirrhosis)
Immunosuppressive drugs (methotrexate, prednisone, cyclosporine)
Malignancy (history of, or active)
Pulmonary insufficiency (room air arterial blood gas < 60%)
Renal failure requiring dialysis
Systemic inflammatory disease (rheumatoid arthritis, systemic lupus erythematosus)
Systemic immune compromise from infection or disease (human immunodeficiency virus, acquired immunodeficiency virus)
Patients with < 1 factors = Systemic host grade A
Patients with 1–2 factors = Systemic host grade B
Patients with > 2 factors = Systemic host grade C

**Table 2:** Pathogens and immediate preoperative inflammatory parameters during initial infection

	No ALS implantation (*n* = 21)	ALS implantation (*n* = 25)
Pathogen detected at initial infection	*Corynebacterium* spp.: *n* = 2	*Corynebacterium* spp.: *n* = 1
	*Enterobacter cloacae*: *n* = 1	*Enterobacter cloacae*: *n* = 1
	*Enterococcus faecium*: *n* = 1	*Enterococcus faecium*: *n* = 1
	*Propionibacterium acnes*: *n* = 1	*Escherichia coli*: *n* = 1
	*Pseudomonas aeruginosa*: *n* = 1	*Propionibacterium acnes*: *n* = 1
	*Staph. epidermidis*: *n* = 2	*Staph. caprae*: *n* = 1
	*Staph. aureus*: *n* = 1	*Staph. epidermidis*: *n* = 3
	MRSA: *n* = 1	*Staph. aureus*: *n* = 1
	No pathogen detected: *n* = 9	MRSA: 7
		Beta-haemolytic *Streptococci*: *n* = 1
		*Viridans streptococci*: *n* = 1
		No pathogen detected: *n* = 7
CRP preoperative (explantation)	Mean: 6.1 mg/dL	Mean: 9,25 mg/dL
		(range: 1,06–41,93 mg/dL)
	(range: 0.72–41.69 mg/dL)	
Leukocyte count	Mean: 12.54 g/L	Mean: 11.56 g/L
		(range: 7.09–19.55 g/L)
	(range: 8.42–21.83 g/L)	
Histopathological results	Positive for infection: *n* = 18	Positive for infection: *n* = 22
	Negative for infection: *n* = 3	Negative for infection: *n* = 3

In one patient of the non-ALS group, the same pathogen (*Staphylococcus epidermidis*) could be detected during re-revision surgery

In all remaining patients (non-ALS and ALS) with additional surgery, no pathogen could be detected

## Statistical analysis

To evaluate the differences in possible confounding parameters between the study group and the control group, the Mann–Whitney *U* test (for numerical and ordinal variables) and the Chi-square test (for binary variables) were applied. Revision-free survival and cumulative survival was calculated using a Kaplan–Meier survival analysis. A log-rank test was applied to detect differences between the observed groups.

Since patients undergoing hip revision arthroplasty show a significant increase in postoperative mortality [24, 25], competing risk analysis was performed to estimate the cumulative incidence function for re-revisions, due to infection accounting for death as a competing event. The cumulative probabilities are given together with the 95% confidence intervals (95% CI). Gray’s test was used to test for statistically significant differences between observed group and *p* values of < 0.05 were considered as statistically significant. Statistical analysis was performed using SPSS software, version 23.0 (SPSS Inc., Chicago, USA) and SAS, version 9.4 (SAS Institute Inc., Cary, NC, USA).

## Results

**Fig. 3:**
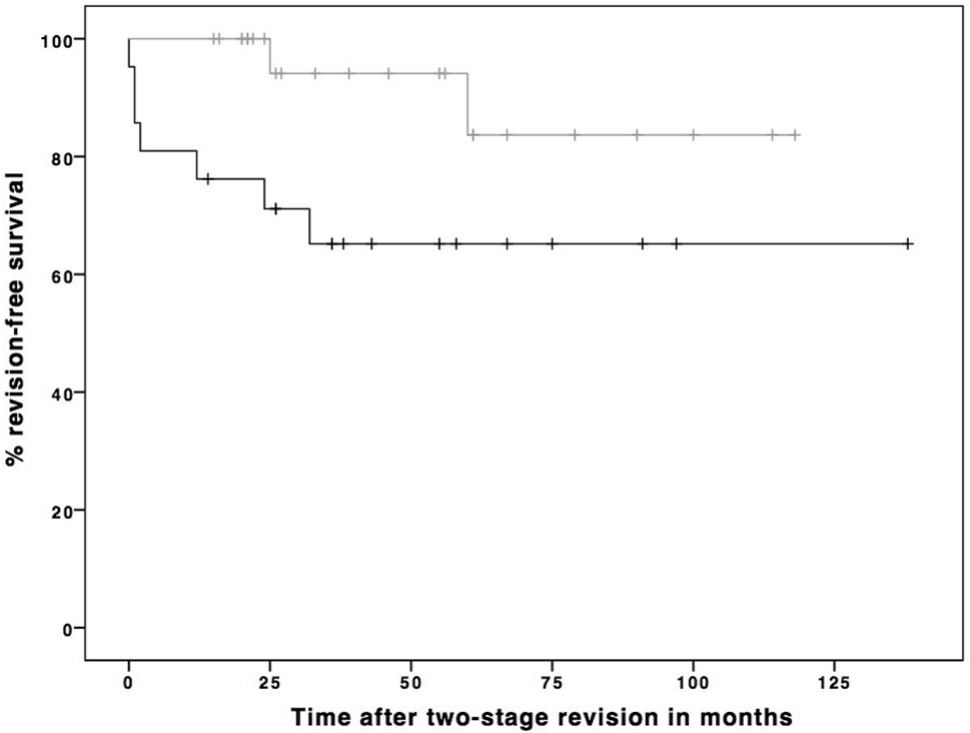
Kaplan–Meier survival curve showing significant (*p* = 0.036) better revision-free survival in patients with interim antibiotic-loaded spacer (gray) compared to patients without an interim antibiotic-loaded spacer (black)

From a total of 46 included patients who received two-stage hip revision surgery, 9 patients (7 non-ALS vs. 2 ALS) had to undergo re-revision surgery due to reinfection of the hip joint. No differences were found in basic demographics between the two groups (Table 3). The mean follow-up was 46 months (range 12–139 months). Kaplan–Meier analysis revealed a revision-free survival in the non-ALS group of 76.1% (*n* = 16) at 12 months, 71.4% (*n* = 15) at 24 months and a long-term revision-free survival rate of 66.6% (*n* = 14). The group with ALS implantation showed a revision-free survival rate of 100% (*n* = 25) at 12 months, 100% (*n* = 25) at 24 months and a long-term revision-free survival rate of 92% (*n* = 23). Log-rank test revealed a significantly better revision-free survival in the ALS group compared to the non-ALS group (*p* = 0.036) (Fig. 3).

**Table 3:** Demographics of patients who received an implantation of an interim antibiotic-loaded cement spacer (ALS) compared to patients without ALS implantation

	No ALS implantation (*n* = 21)	ALS implantation (*n* = 25)	*p value*
Age (mean)	61 (± 15.5)	67 (± 12.1)	*0.15*
Gender	Female: 52.4% (*n* = 11)	Female: 40%(*n* = 10)	*0.553*
	Male: 47.6% (*n* = 10)	Male: 60% (*n* = 15)	
Systemic host grade [8]	A: 28.6% (*n* = 6)	A: 36% (*n* = 9)	*0.424*
	B: 61.9% (*n* = 13)	B: 44% (*n* = 11)	
	C: 9.5% (*n* = 2)	C: 20% (*n* = 5)	
Infection type [8]	Early (< 4 wks): 9.5% (*n* = 2)	Early (< 4 weeks): 4% (*n* = 1)	*0.585*
	Hematogenous (< 4 wks): 0%	Hematogenous (< 4 weeks): 0%	
	Late (≥ 4 wks): 90.5% (*n* = 19)	Late (≥ 4 weeks): 96% (*n* = 24)	

Systemic host grade A represents patients without any compromising host factors (comorbidities), grade B involves patients with 1–2 compromising factors and grade C equals > 2 compromising factors

Compromising Host Factors are listed in Table 1

**Fig. 4:**
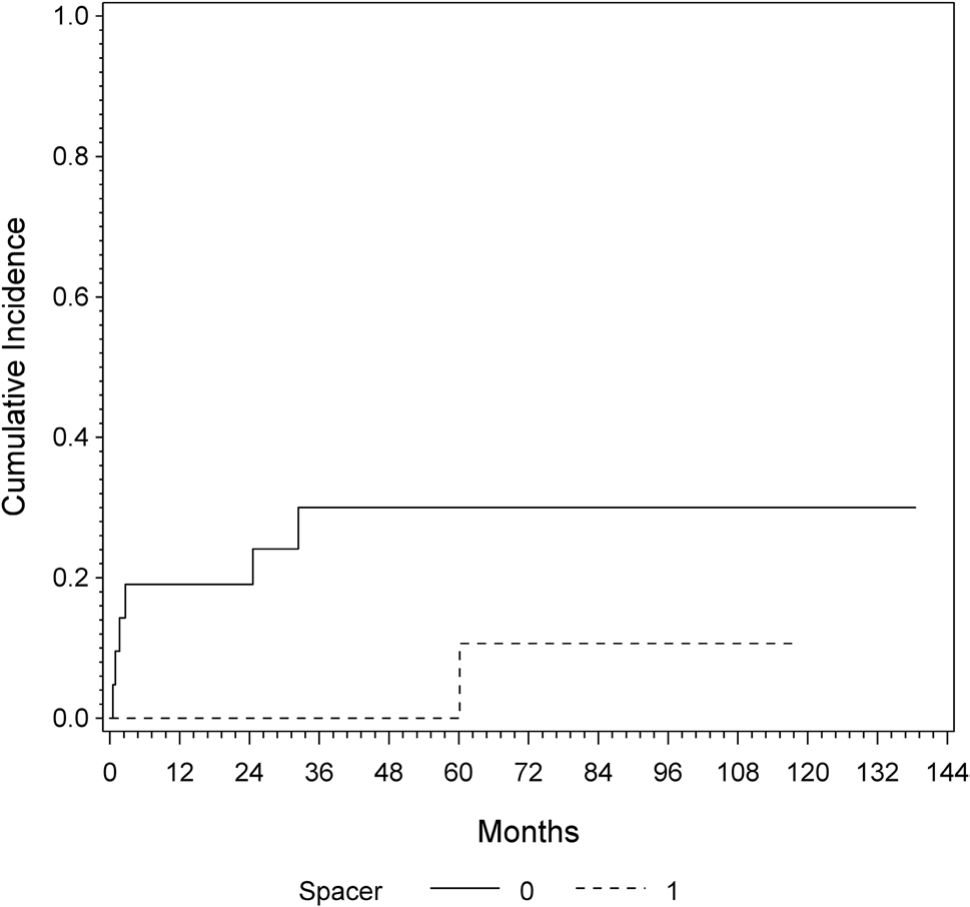
Cumulative incidence of re-revision in patients with implantation of antibiotic-loaded spacer (dotted) and without interim antibiotic spacer (line), assessed by competing risk analysis

After reimplantation, one patient of the ALS died 16 months after reimplantation. In the non-ALS group, two patients died 15 and 91 months, respectively, after reimplantation. Therefore, for the ALS cohort, an estimation of cumulative incidences revealed an overall risk of re-revision surgery after the second stage of 8.7% (95% CI 3–19%) at 12 (42 patients followed-up) and 24 months (33 patients followed-up) and 14.6% (95% CI 6–28%) at 36 months (24 patients followed-up). The non-ALS group showed a risk of re-revision of 19% (95% CI 5–38%) at 12 and 24 months and 30% (95% CI 12–51%) at 36 months. The group with ALS implantation displayed a risk of re-revision surgery of 0% in the first 36 months. At 60 months (nine patients followed-up), the risk of re-revision increased to 10% in this group. The Gray test revealed a significant difference in the cumulative incidence between both observed groups (*p* = 0.026) (Fig. 4).

In regard to the aforementioned “difficult-to-treat” (DTT) microorganisms, seven patients in the ALS group were initially infected with DTT of whom six patients suffered from an infection with Methicillin-resistant *Staphy. aureus* (MRSA), and in one patient MRSA and *Enterococcus faecalis* was found. One of those seven patients had to undergo additional revision surgery and further antibiotic treatment due to reinfection. Reduced susceptibility to Vancomycin could not be detected in this case. In the group without ALS implantation, one patient suffered from MRSA and another patient from *E. faecalis* infection. Both patients had to undergo re-revision surgery, but no pathogen could be detected during these additional revision surgeries. Only in one patient of the non-ALS group, the same pathogen (*Staphy. epidermidis*) could be detected during re-revision surgery. In all remaining patients (both ALS and non-ALS) with additional surgery, no pathogen could be detected.

## Discussion

Two-stage revision surgeries represent a standard procedure for the treatment of periprosthetic joint infections (PJI). Antibiotic-loaded cement spacers (ALS) are broadly used as an interim replacement for the interval between explantation and reimplantation. ALS implantation not only leads to higher local antibiotic-levels between first and second stage surgery [26], it also improves the functional and satisfactory outcome in terms of an improved joint mobility and a lower contracture rate [27]. ALSs also facilitate reimplantation due to minimizing leg length discrepancy [28, 29]. Nevertheless, concern still exists whether ALS should be implanted in two-stage revisions by default [9, 19, 30, 31].

We, therefore, raised the question of if ALS implantation reduces the risk of reinfection after two-stage revisions due to periprosthetic hip infection.

After thorough research on this topic, this is the first single-center study to compare two-stage hip revisions with or without interim ALS implantation in regard to the risk of reinfection.

The fact that the use of an interim ALS declined the re-revision rate and improved the outcome with regards to a reduced risk of reinfection represent major findings in this study. From our perspective, the use of ALS in two-stage hip revisions is recommended. These recommendations are supported by the fact that besides establishing the use of ALS in two-stage hip revision cases in our department in 2008, no further changes regarding surgical approach and systemic antibiotic treatment have been perceived during the whole observational period between both groups in this retrospective cohort study. Therefore, it seems conceivable that the decline in the re-revision rate and reduced risk of reinfection is due to the use of ALS.

In the literature, two-stage revisions deliver the highest success rate with re-revision rates of about 10% [32]. Despite a high overall re-revision rate of 19.6%, ALS implantation decreased revisions after two-stage exchange and lead to a long-term implant survival rate of 92%. These results confirm the findings of Chen et al., where they gathered similar reinfection rates in two-stage revisions with ALS [33].

In spite of this, ALS represents a foreign body that is implanted in the infected joint. This may lead to a slowdown, or even an inhibition of PJI eradication [34]. Therefore, some authors discourage the non-restrictive use of ALS [22, 34, 35].

In 2015, Gomez et al. investigated the outcome of two-stage revisions in the treatment of periprosthetic infection [15]. They found that a substantial amount of patients do not undergo reimplantation after spacer implantation and about one-fifth of patients needed revision after reimplantation. In our cohort, only one patient underwent spacer exchange due to persistent infection and the overall re-revision rate corresponds to the results by Gomez et al. However, static and articulating spacers were included in their study, whereas only articulating spacers were used in our investigation, and articulating spacers seem to produce better results than static spacers [36, 37].

Zimmerli et al. state that surgeons should refrain from using ALS implantation in cases with “difficult-to-treat” (DTT) microorganisms. Reason for this restriction is that the ALS itself may represent a pathogen vehicle, especially in cases caused by antibiotic-resistant pathogens. The ALS may then eventually contribute to biofilm formation and the treatment may become more complex and difficult. These DTT microorganisms involve Methicillin-resistant *Staphy. aureus* (MRSA), small colony variants of staphylococci, enterococci, quinolone resistant *P. aeruginosa* and fungi. [19, 22]. Due to the low number of DTT cases in this study, we cannot give distinct evidence to discourage the reader from this recommendation. Nonetheless, we still found a tendency for a better outcome for DTT infections with ALS implantation. However, studies with larger sample sizes are needed to confirm this statement.

This study shows some limitations: First, due to its retrospective character and the long observation period, some selection bias can be expected. This selection bias may be due to the fact that 17 out of the 21 patients who did not receive ALS were treated between 2001 and 2008, and 21 out of 25 patients with ALS implantation were treated after 2008. Therefore, the better outcome of the ALS group may also be due to the fact that surgical techniques and antibiotic treatment improved during the observation period. Second, the relatively low number of patients may lead to an over- or underestimation of the results, but our sample size seems similar with other studies dealing with this subject, and therefore, our results remain comparable.

## Conclusion

Our findings suggest that ALS implantation reduces the risk of reinfection after two-stage hip revision surgery. These results support the position that AB spacers fulfil the requirements of effective AB levels and seem to achieve a broad bacterial coverage. However, larger studies are needed to confirm this statement for infection with pathogens, which are known as difficult to treat. Additionally, since the development of AB resistances and the occurrence of biofilm-forming microorganisms increase, further investigations regarding new developments in diagnostics and treatment strategies should be obtained constantly.
